# Raclopride-Molecularly Imprinted Polymers: A Promising Technology for Selective [^11^C]Raclopride Purification

**DOI:** 10.3390/ma16031091

**Published:** 2023-01-27

**Authors:** Roberta Del Sole, Giancarlo Pascali, Giuseppe Mele, Gary Perkins, Lucia Mergola

**Affiliations:** 1 Department of Engineering for Innovation, University of Salento, 73100 Lecce, Italy; 2 School of Chemistry, The University of New South Wales, Sydney, NSW 2052, Australia; 3 Australian Nuclear Science and Technology Organisation, Sydney, NSW 2234, Australia

**Keywords:** molecularly imprinted polymer, raclopride, radiotracer, solid phase extraction, positron emission tomography, HPLC

## Abstract

In this work, we developed a novel approach to purify [^11^C]Raclopride ([^11^C]RAC), an important positron emission tomography radiotracer, based on tailored shape-recognition polymers, with the aim to substitute single-pass HPLC purification with an in-flow trap & release process. Molecular imprinting technology (MIT) applied to solid phase extraction (MISPE) was investigated to develop a setting able to selectively extract [^11^C]RAC in a mixture containing a high amount of its precursor, (S)-O-Des-Methyl-Raclopride (DM-RAC). Two imprinted polymers selective for unlabeled RAC and DM-RAC were synthesized through a radical polymerization at 65 °C using methacrylic acid and trimethylolpropane trimethacrylate in the presence of template molecule (RAC or DM-RAC). The prepared polymer was characterized by scanning electron microscopy and Fourier transform infrared spectroscopy and tested in MISPE experiments. The polymers were used in testing conditions, revealing a high retention capacity of RAC-MISPE to retain RAC either in the presence of similar concentrations of RAC and DM-RAC precursor (96.9%, RSD 6.6%) and in the presence of a large excess of precursor (90%, RSD 4.6%) in the loading solution. Starting from these promising results, preliminary studies for selective purification of [^11^C]Raclopride using this RAC-MISPE were performed and, while generally confirming the selectivity capacity of the polymer, revealed challenging applicability to the current synthetic process, mainly due to high backpressures and long elution times.

## 1. Introduction

Positron emission tomography (PET) represents an extremely powerful diagnostic imaging technique that permits the specific identification of several pathologic conditions, such as cancer, heart failures, and brain disorders. In this approach, radioactive tracers are used to reveal the underlying molecular functionalities active in pathological disorders [1]. The unsurpassed capability of PET radiopharmaceuticals to inspect biochemical pathways in a functional way, yet without modifying the normal homeostasis of the studied organisms, is an important tool in molecular imaging. This potential, with valuable consequences in drug discovery, biological studies, and diagnosis just to cite a few fields, is, however, blunted by the scarce availability of a wide range of tracers able to specifically image different pathologies. This fact is due to the difficult chemistry involved (and therefore the limited availability of skilled scientists) and the time constraints linked to the decay features of most PET nuclides (i.e., short half-life). Especially this time constraints are crucial, not merely for the need for quick and efficient radiochemical procedures, but also for the requirement of absolutely reliable and repeatable processes, since the tracer production must happen in exactly the same way every time the imaging experiment is planned (regardless operator, site, machine set-up, and other logistics parameters). Many radiotracers used in PET include in their structures radioactive versions of naturally occurring elements such as ^11^C, ^18^F, ^13^N, and ^15^O, thus allowing a mimetic labeling approach. [^11^C]raclopride ([^11^C]RAC) is the most important dopamine D2-like antagonist, widely used as a radiotracer in PET [2,3,4,5] to monitor psychiatric and neurologic disorders such as schizophrenia, Huntington’s Chorea, and Parkinson’s disease [3], in which an alteration of Dopamine D2 receptors occurs [6]. For this reason, it is fundamental to monitor their functionality and availability, to better assess the treatment options and follow the patients over their course [2,7].

**Figure 1:**
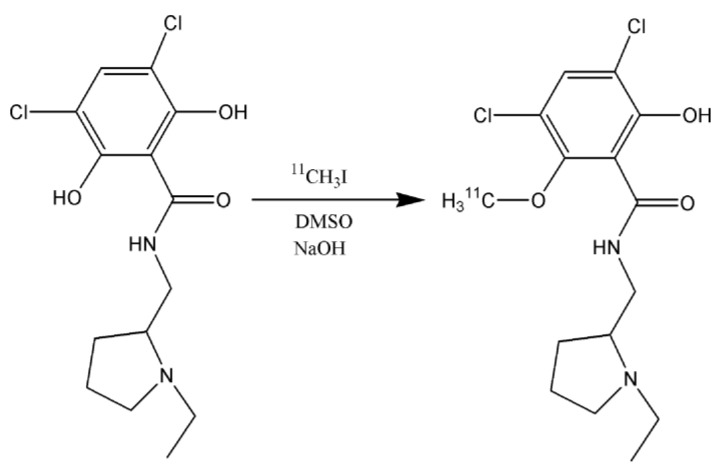
Synthesis of [^11^C]RAC starting from the free base precursor DM-RAC and ^11^CH_3_I.

Generally, [^11^C]RAC is synthesized by methylation of (S)-O-Desmethyl Raclopride (DM-RAC) precursor with [^11^C]methyl iodide (^11^CH_3_I) or [^11^C]methyl triflate through traditional vial-based approach or using solid support such as cartridge or loop [1,6,8], with the aim to obtain highly controllable, yet flexible, production processes (Figure 1).

However, the majority of the efforts have been focused on the reactive steps (e.g., radiolabeling) [8], while the purification and formulation are still performed in the traditional ways, e.g., HPLC, SPE, and solvent exchange [9,10]. This is not ideal, especially in the case of highly productive reactive modalities (e.g., microfluidics, microwaves, photochemistry), for which the reactions can be completed in a few tens of seconds, while the traditional purification/formulation approach can take up to 45 min, thus defeating the prospected time gain, with also solvent waste and final formulation steps. Therefore, the development of a fast, simple, and selective purification technique is an important goal. In this respect, molecular imprinting technology (MIT) is a viable synthetic approach to design highly selective molecular recognition elements able to specifically recognize a target molecule. The use of such a concept is not new to radiochemistry, and it has been previously reported to clean up radiofluorination mixtures before performing the next synthetic step [11]; however, this example remains isolated and not widely adopted.

MIT permits the production of a specific polymer through the copolymerization of a functional monomer with a target analyte (template) in the presence of a large excess of cross-linking agents. After polymerization, the template is removed with several washing steps, leaving in the polymer-specific recognition sites that are complementary in size and shape to the template. In this way, an intelligent synthetic system with high selectivity towards the template can be generated [12,13,14,15,16]. The imprinted polymer obtained, compared to the biological system, often used for its high recognition capacity, has inertness towards acidic, alkaline, and organic solvents but also high robustness and resistance to elevated temperature and pressure. In addition, the storage life of imprinted polymers can be very high (several years at room temperature), and after washing can be reused many times without losing their recognition ability. These important properties make molecularly imprinted polymers (MIPs) suitable for their use in a lot of application fields, such as chemical separation, catalysis, and electrochemical sensing [11,15,17,18].

The present work is focused on the development of a novel purification approach for [^11^C]RAC that will exploit specifically tailored shape-recognition polymers with the aim to substitute the standard single-pass HPLC purification with an in-flow trap and release sequence. In particular, MIT applied to solid phase extraction (MISPE) [14,18,19,20] was studied in order to develop a setting able to selectively extract [^11^C]RAC in a mixture containing a high amount of its precursor, DM-RAC, a condition that occurs after [^11^C]RAC radiolabeling step. To prepare two MIPs able to bind unlabeled RAC and DM-RAC, a thermal polymerization was used. The obtained polymers were packed into empty SPE cartridges and evaluated as selective sorbents for RAC purification from RAC/DM-RAC mixtures. The newly developed protocol that proved able to purify RAC molecules was then tested using in-flow trap and release systems to verify the applicability of this concept to radiopharmaceutical manufacturing.

## 2. Materials and Methods

### 2.1. Materials and Apparatus

All chemicals were of analytical grade or the highest purity available. Unlabeled RAC [119,670-11-0] and DM-RAC [84,225-95-6] were supplied from ABX advanced biochemical compounds GmbH (Radeberg, Germany). Methacrylic acid (MAA) [79-41-4] and α-α′-azoiso-butyronitrile (AIBN) [78-67-1] were purchased from Fluka (Steinheim, Germany). Analytical grade acetonitrile (CH_3_CN) [75-05-8], methanol (CH_3_OH) [67-56-1] and ethanol (EtOH) [64-17-5] were supplied from J.T. Baker (Deventer, The Netherlands). Trifluoroacetic acid (TFA) [76-05-1], formic acid [64-18-6], sodium acetate (NaOAc) [127-09-3], acetic acid (AcOH) [64-19-7], dimethyl sulfoxide (DMSO) [67-68-5], sodium hydroxide (NaOH) [1310-73-2] and trimethylolpropane trimethacrylate (TRIM) [3290-92-4] were obtained from Sigma-Aldrich (Steinheim, Germany). All solutions and buffers were prepared with ultrapure water obtained with a water purification system (Human Corporation, Seoul, Korea). Chromabond empty SPE cartridges (3 mL) was supplied from Macherey-Nagel (Duren, Germany), while Valco Fingertight HPLC Cartridge Columns were obtained from Vici (Brockville, ON, Canada).

The automated radiochemical reactions were performed using a Synthra MeI coupled with a GPextent (Synthra GmbH, Hamburg, Germany), as previously described [6]; whereas indicated, the MISPE system (packed in a metal cartridge or glass Omnifit column) were placed in place of the original HPLC column.

Sonication was carried out using a Sonorex RK 102 H ultrasonic water bath from Bandelin Electronic (Berlin, Germany). Centrifugation was achieved with a PK121 multispeed centrifuge from Thermo Electron Corporation (Château Gontier, France). UV-visible spectra were recorded using a Jasco V-660 UV-visible spectrophotometer (Jasco, Palo Alto, CA, USA). HPLC analysis was conducted using an Agilent 1100 Series Liquid Chromatography system coupled to a DAD using a C18 column (150 mm × 4.6 mm i.d., 5 μm SS Wakosil C18 column) thermostated at 25 °C. The mobile phase was composed of an acidic aqueous solution (formic acid 1%) (solvent A) and CH_3_CN containing 1% of formic acid (solvent B) at a flow of 0.5 mL min^−1^. The following gradient was used: 0 min, 30% B; 9 min, 50% B; 14 min 50% B, STOP. All chromatograms were acquired at 212 nm for RAC and 279 nm for DM-RAC. For quantification, reference standard solutions containing known amounts of RAC and DM-RAC were analyzed and the peak areas versus the concentration were plotted. For radiochemical experiments, a Shimadzu system comprising a CBM-20 controller, LC-20AD pump, SIL-20AHT auto-injector SPD-M20A PDA (UV-220 nm), and a Lablogic Posi-RAM gamma detector was used (Shimadzu, Rydalmere, Australia). Solvent mixture was passed through a Shimadzu online degasser DGU-20A (Rydalmere, Australia). Analysis conditions used were the following: Phenomenex Kinetex column C8 (50 mm × 4.6 mm, 2.6 µm) with mobile phase 35% CH_3_OH, 65% 20 mM ammonium acetate (pH = 4.5) at a flow rate of 1 mL min^−1^. For pH measurements, a pHmeter Basic 20 was used (Crison Instruments, Barcelona, Spain). Scanning electron microscopy (SEM) images were conducted with a Phenom ProX microscope (Phenom-World B.V., Eindhoven, The Netherlands) equipped with a high-sensitivity backscattered electron detector that allows compositional and topographical image modes. A JASCO 660 plus infrared spectrometer (Easton, MD, USA, www.jascoinc.com, accessed on 22 December 2022) was used for FTIR analysis. Dry polymer was directly placed on an ATR PRO450-S single reflection ATR accessory. Liquid MAA was spread directly on an ATR ZnSe crystal.

### 2.2. Preparation of Molecularly Imprinted Polymer Selective for Unlabeled Raclopride and (S)-O-Des-Methyl-Raclopride

MIPs for unlabeled RAC and DM-RAC, RAC-MIP, and DM-RAC-MIP, respectively, were prepared by bulk polymerization using CH_3_CN as porogen solvent. Briefly, the template and the functional monomer (MAA) were put in a glass tube and dissolved in about 2 mL of CH_3_CN. To facilitate the dissolution of DM-RAC in CH_3_CN, 8 µL TFA was added. After addition of TRIM, the solutions were sonicated for 5 min. The molar ratio between template, functional monomer, and cross-linking agent was 1:4:20. To start the reaction, AIBN was added to the solution, purged with nitrogen gas, and sonicated for 5 min. The polymerization process was carried out at 65 °C for 24 h. Successively the polymers obtained were ground and sieved in the range of 20–70 µm. All polymers were washed several times with CH_3_OH to remove any unreacted materials and subsequently washed with CH_3_OH/AcOH (7/3, *v*/*v*) to remove the template molecule. Finally, polymeric particles were washed with double distilled water to obtain a neutral pH, and the resulting fine powders were dried under a vacuum in a desiccator. The control polymers (NIPs) were synthesized under the same conditions but in the absence of the template molecules. A preliminary evaluation of binding performances was made by batch rebinding experiments, incubating 10 mg of each polymer (MIPs and NIPs) with known concentrations of RAC and DM-RAC (0.3–35 mg L^−1^) dissolved in 2 mL of the porogen solvent. Then, a comparison between the adsorption capacities of MIPs and NIPs was made, and the imprinting factor (IF) was calculated as reported in a previous work [21]. Then, 150 mg of dry polymers were packed in empty SPE cartridges between two polyethylene frits.

### 2.3. MISPE for Unlabeled Raclopride Extraction

The cartridge packed with RAC-MIP (RAC-MISPE) was conditioned with 4 mL of acidified aqueous solution (AcOH 0.1%). To mimic the synthesis conditions in the radiochemical process, one milliliter of aqueous solution of unlabeled RAC and DM-RAC with 5.3% of DMSO and trace of NaOH 5 M (0.3%), was loaded onto the cartridge. RAC-MISPE cartridge was conditioned with 4 mL of acidified aqueous solution with 0.1% of AcOH to activate polymeric particles. To remove DM-RAC, selective washing using a mixture of buffer acetate 20 mM (pH 5) and EtOH (1/1, *v*/*v*) was used. After a series of washing steps with ultrapure water to remove residual buffer solution, the intramolecular conditions of the cartridge were modified using 2 mL of NaOH 0.025 M (reconditioning step). Afterward, RAC was eluted using 5 mL of H_2_O/EtOH (*v*/*v*, 9/1) to obtain RAC in a biocompatible solution. After each experiment, the cartridge can be regenerated using 6 mL of CH_3_CN and 4 mL of ultrapure water and reused indefinitely. A second attempt was also made using a cartridge packaged with DM-RAC-MIP (DM-RAC-MISPE). In this case, the cartridge was conditioned with 5 mL of NaOH 5 M (0.3%) and then loaded with one milliliter of aqueous solution of unlabeled RAC (10 µg mL^−1^) and DM-RAC (10 µg mL^−1^) in the presence of DMSO (5.3%) and trace of NaOH 5 M. Then the cartridge was washed with 2 mL of NaOH 5 M and finally DM-RAC was eluted with 2 mL of acetate buffer 20 mM (pH 5). All fractions were analyzed by HPLC and, starting from the peak area, the recovery yield (%) was calculated as the difference between the initial amount of molecule loaded on the cartridge and the amount of molecule identified in the fraction.

The selectivity coefficient *k* of RAC, relative to the competing DM-RAC, was calculated using the following equation:

(1) $$k=\frac{\mu g\;RAC_{retained}}{\mu g\;DM-RAC_{retained}}$$

where *μg RAC_retained_* and *μg DM-RAC_retained_* represent micrograms of RAC and DM-RAC calculated considering the difference from micrograms of RAC and DM-RAC loaded on the cartridge and micrograms of RAC and DM-RAC found in the waste (loading, washing and reconditioning fractions).

To evaluate the specificity of RAC-MIP, a cartridge was also packed with the corresponding NIP (RAC-NIP) and the same experimental procedure used for RAC-MISPE was adopted to estimate the retention of RAC.

### 2.4. Implementation of MISPE Cartridge in “in-Flow Trap & Release System” for [^11^C] RAC Purification

**Figure 2:**
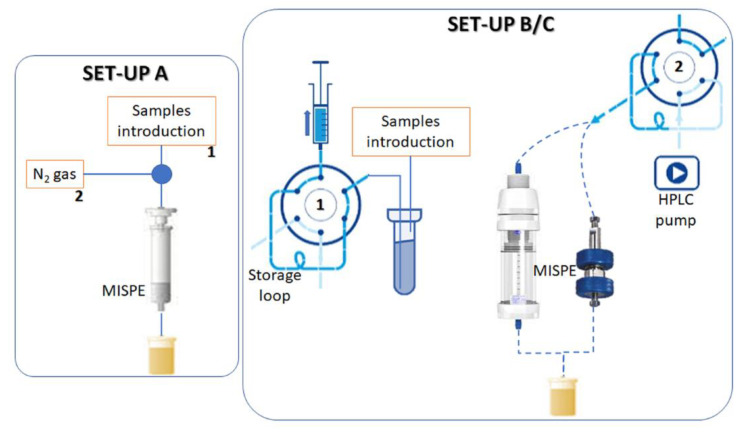
Schematic representation of the 3 set-ups employed. In all set-ups, chemicals are first loaded in the MISPE (step **1** in the scheme) and then eluted out of the MISPE (step **2** in the scheme). Set-up B and C differ from the type of cartridges used to contain the MISPE: Valco fingertight HPLC column (**right**, set-up B) and Omnifit glass column (**left**, set-up C).

Three different set-ups were employed for testing the performance of RAC-MISPE for [^11^C]RAC purification (Figure 2). Firstly (**set-up A**), the same plastic cartridge used for non-radioactive tests was placed in a shielded fume hood, with solutions added manually and liquids pushed by nitrogen pressure (up to 5 bar). This setup was used to trap and release a solution of [^11^C]RAC already purified by standard semi-prep HPLC. The polymer was previously activated, as indicated above (AcOH 0.1%). After loading the radioactive product, since there was no DM-RAC to separate, the polymer was washed with 2 mL of H_2_O and prepared for elution with 2 mL of 5 M NaOH; the elution was performed with 2 portions of 2 mL of pure EtOH. This setup allowed the measurement of radioactive content of both MISPE and retrieved solution at each step.

To test the capacity of such system to replace single-pass HPLC purification, we devised a different setting (**set-up B**), in which we filled a Valco metal cartridge with the polymer and placed it instead of the HPLC column. We then added in different reservoirs the solutions needed for dilution (H_2_O, 2 mL), DM-RAC cleaning (0.025 M NaOAc/EtOH 1/1, 8 mL), reconditioning (5 M NaOH, 2 mL) and elution (EtOH, 4 mL); such solutions were loaded in a 10 mL SS injection loop and pushed in the MISPE cartridge by the HPLC pump as metering pump, using H_2_O as inert carrier. The whole radiolabeling mixture was employed, and its starting amount of radioactivity was estimated (i.e., Synthra radiodetector) before passage through MISPE. This setup allowed us to monitor the system backpressure and to measure the radioactive content of retrieved solutions at each step; no direct assessment was achievable on the MISPE cartridge, given the impossibility to retrieve it safely from an area of high radioactive background.

In an attempt to reduce the system backpressure, a similar setting (**set-up C**) was devised, by changing the Valco metal cartridge with an Omnifit glass column with adjustable height and performing the same separation process.

## 3. Results and Discussions

Perkins and co-workers optimized the radiochemistry condition for [^11^C]RAC production using a Synthra GPextent system starting from ^11^CH_3_I and free bases precursor (DM-RAC) through a loop method in the presence of DMSO as reaction solvent and traces of NaOH. In this process, the radiolabeling mixture is composed of traces of desired product in the presence of a large excess of des-methyl precursor. Since the precursor also features some affinity for D2 receptors, the mixture requires purification, currently achieved by single-pass semi-preparative HPLC [6].

### 3.1. Optimization of MIP Performance with Non-Radioactive Standards

Starting from Del Sole and co-workers’ expertise in the field of imprinting technology [12,13,16,19,21,22] and using the labeling reaction composition as starting knowledge, the design of a purification method of [^11^C]RAC employing molecularly imprinted polymeric particles was considered advantageous in terms of simplicity and reliability of radiopharmaceutical manufacturing. Assuringly, in recent years several groups have published numerous works demonstrating the use of MIT in the preparation of selective systems for the extraction of important compounds [13,15,18,23,24]. Therefore, we prepared MIPs selective for [^11^C]RAC and DM-RAC using non-radioactive templates and standard approaches. Typical methacrylate polymers were obtained by radical polymerization of methacrylic acid in the presence of the template by using TRIM as a cross-linking agent in order to obtain a highly cross-linked structure. The resulting RAC imprinted polymer was characterized by SEM to assess useful morphological features such as surface structure and particle shape and to confirm polymer formation.

**Figure 3:**
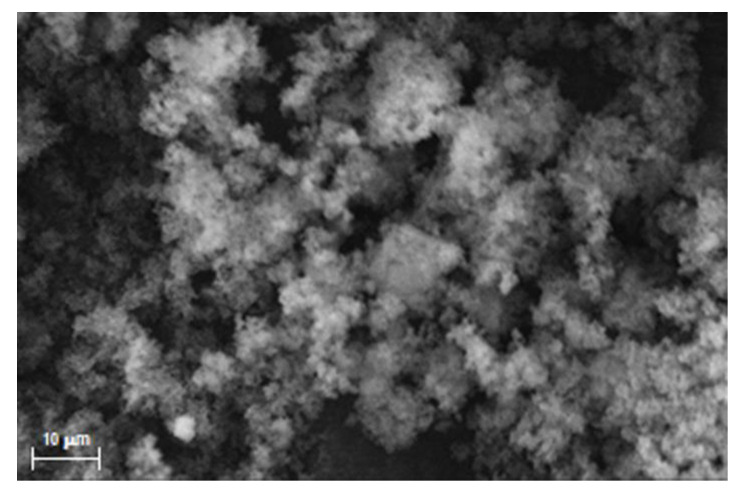
SEM image of RAC-MIP (3000× magnification).

As shown in Figure 3, an amorphous structure with the presence of polydisperse aggregates with irregular shapes was observed, as expected using a bulk polymerization for similar MIP systems, due to the high amount of the cross-linker added during the polymerization process [15,23,25,26]. Similar results were also obtained by analyzing SEM images of DM-RAC.

**Figure 4:**
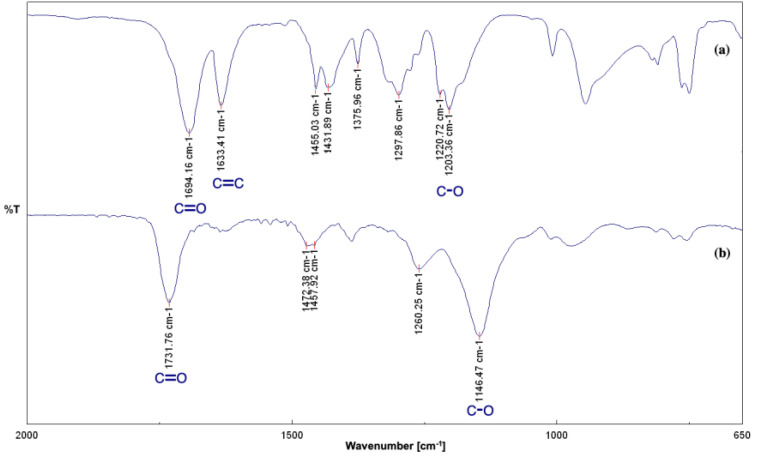
FTIR analysis of (**a**) MAA and (**b**) RAC-MIP.

Synthesized MIPs were also analyzed by FTIR analysis (Figure 4).

As can be seen in Figure 4, MAA (Figure 4a) and RAC-MIP (Figure 4b) spectra were reported. FTIR spectrum of MIP shows the typical bands of a polymer prepared from MAA and TRIM, functional monomer, and crosslinker, respectively [14]. A strong signal at 1731 cm^−1^ is attributable to the stretching of the C=O band of the carboxylic acid with the loss of conjugation of MAA in the polymeric material (Figure 4b), confirmed by the disappearance of the peak at 1633 cm^−1^ typical of the C=C, as can be seen in MAA spectrum. In fact, in the MAA spectrum (Figure 4a), a typically strong peak at 1694 cm^−1^ for the C=O stretching of the conjugated carboxylic acid can be observed that it moved at 1731 cm^−1^ in the polymer spectrum due to the absence of the conjugation; a peak at 1633 cm^−1^ typical of the C=C stretching. Moreover, it can be observed a signal around 1455 cm^−1^, due to the C-O-H bending and CH_2_ bending, and a signal at 1203 cm^−1^, ascribable to the C-O stretching of the carboxylic acid.

After characterization, a preliminary evaluation of MIPs and NIPs binding performances was made through batch rebinding experiments, incubating all polymers with RAC and DM-RAC solutions at different concentrations. Adsorption capacities of RAC-MIP and DM-RAC-MIP showed the presence of a plateau around 5–6 mg g^−1^. Then, the IF was calculated as a ratio between the adsorption capacities of each MIP with the corresponding NIP at a known concentration (28 mg L^−1^). The results obtained showed an IF of RAC-MIP and DM-RAC-MIP equal to 15.8 and 12.4, respectively.

RAC and DM-RAC imprinted polymers were packed in SPE cartridges and tested to evaluate the best conditions to obtain RAC purification, also in the presence of a large excess of DM-RAC.

A first experiment was conducted by loading on the RAC-MISPE cartridge 1 mL of an aqueous solution (5.3% of DMSO and trace of NaOH 5 M) containing the same amount of RAC and DM-RAC (10 µg mL^−1^). The solvent used mimics the synthesis condition employed for labeled RAC production. Moreover, a low concentration of RAC was chosen in order to use a similar RAC concentration present in labeled RAC production. The pH of the loading solvent is around 12, and in this environment, RAC and DM-RAC present probably a mono-anionic and dianionic conformation, respectively, due to the deprotonation of hydroxyl groups present in their structures.

**Figure 5:**
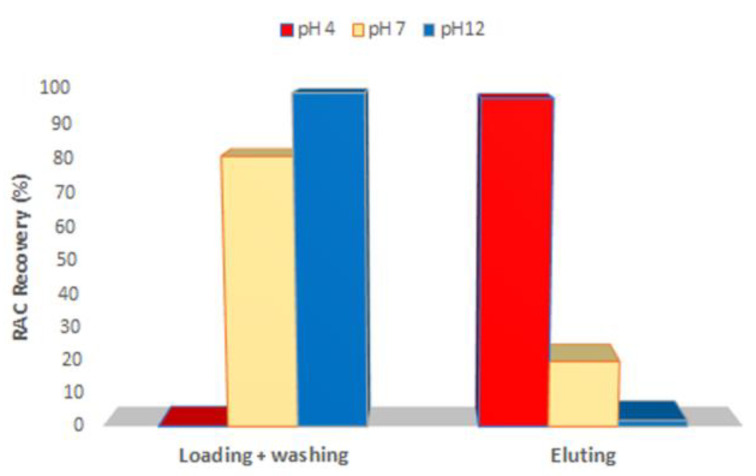
RAC recoveries obtained from RAC-MISPE using different pH of conditioning solvent.

To evaluate the best condition for RAC retention, the RAC-MISPE was firstly conditioned with ultrapure water (pH 7), but no interaction was observed. For this reason, several attempts were made to modify the pH of the solvent to condition the cartridge. It was seen that the presence of an acidic environment (AcOH 0.1%) with a pH of around 4 created the right interactions to promote the retention of RAC (Figure 5).

**Figure 6:**
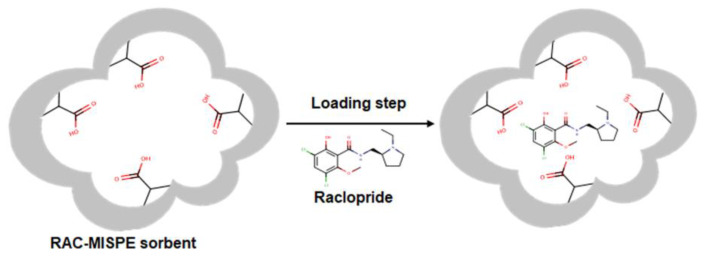
Schematic representation of interaction between RAC and RAC-MISPE sorbent.

In particular, the presence of an on/off system that activates and deactivates the retention of RAC, depending on the pH, was noted. Conditioning the cartridge with 0.1% acetic acid, polymeric particles were activated to retain the template RAC strongly and selectively, also in the presence of its precursor DM-RAC. Furthermore, RAC imprinted polymer features specific cavities and functional groups positioned in a well-defined spatial arrangement reflecting the tridimensional structure of RAC, promoting a much stronger interaction of this compound with the polymer compared to DM-RAC (Figure 6). Indeed, the main advantage of MIT is to discriminate between very similar molecules that differ only for their spatial conformation (chiral molecules) [27,28,29] or small functional groups [13,14,30,31]. In this case, DM-RAC differs structurally from RAC only in the presence of a proton instead of a methyl group.

**Figure 7:**
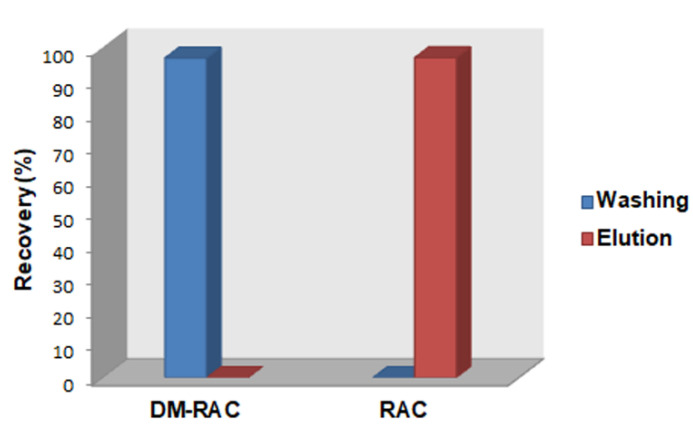
Recovery yield in washing and elution steps for DM-RAC (10 µg mL^−1^) and RAC (10 µg mL^−1^) loaded on RAC-MISPE cartridge packed with RAC-MIP.

During the washing steps, conducted using a mixture of buffer acetate and EtOH (1/1, *v*/*v*), RAC was strongly retained, while a complete release of its precursor DM-RAC was observed. In order to elute RAC, the polymer needs to be deactivated by modifying the pH of the system. For this reason, 2 mL of NaOH 0.025 M (reconditioning solution) was loaded on the cartridge. These conditions favor the deprotonation of RAC and consequently break the hydrogen bonds, reducing polymer interactions and, in consequence, facilitating RAC elution. Therefore, elution of RAC was achieved using 5 mL of H_2_O/EtOH (*v*/*v*, 9/1) to obtain RAC in a biocompatible solution. As can be seen in Figure 7, during the washing steps, the precursor was removed (96.7%, RSD 1.2%), and about 96.9% (RSD 6.6%) of unlabeled RAC was eluted in the final step with a high grade of purity due to the complete absence of DM-RAC (Figure 7). These results confirmed a high selectivity of RAC-MIP for RAC that was selectively retained also in the presence of DM-RAC. Indeed, the selectivity coefficient (*k*) calculated was very high (193.4).

**Figure 8:**
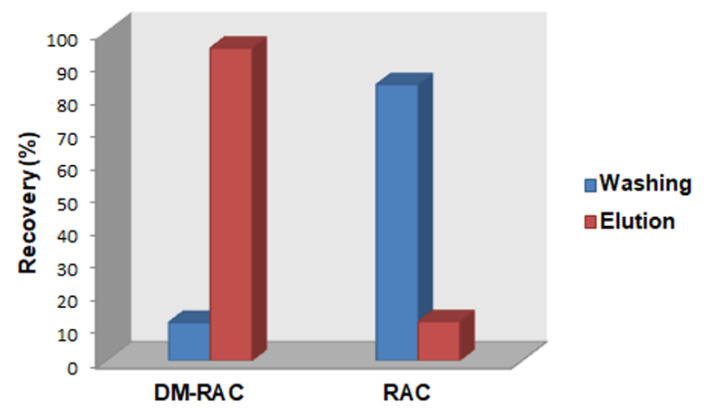
Recovery yield in washing and elution steps for DM-RAC (10 µg mL^−1^) and RAC (10 µg mL^−1^) loaded on DM-RAC-MISPE cartridge packed with DM-RAC-MIP.

Successively, the second cartridge DM-RAC-MISPE was tested with the aim to retain DM-RAC, favoring the purification of RAC using a “pass-through” approach that would be extremely fast and attractive. Starting from the consideration made before on best condition favoring the bind and the elution of RAC from the polymer, the cartridge was conditioned using a solution of NaOH 5 M (0.3%) to modify the chemical environment of the polymer, favoring the washing of RAC and the retention of DM-RAC. Basic conditions promote the deprotonation of RAC but also of functional groups present in the polymer, considerably reducing the unspecific bonds that could occur between RAC and polymeric particles. Afterward, the cartridge was loaded with the same solution mentioned before and washed with 2 mL of NaOH 5 M (0.3%), which led to the removal of 83.8% (RSD 3.6%) of RAC but also of 11.6% (RSD 1.5%) of DM-RAC. After the elution made using 2 mL of acetate buffer 20 mM (pH 5), 94.8% (RSD 1.9%) of DM-RAC was eluted, but in the presence of 11.9% (RSD 3.5%) of RAC (Figure 8).

In this case, DM-RAC-MIP showed minor selectivity towards DM-RAC, as demonstrated by the low value of *k* obtained (7.9). Comparing the results obtained by using the two different MIP sorbents, it is evident that the RAC-MISPE cartridge showed the best performance and permits better separation of RAC and its elution in a biocompatible mixture.

To ensure the specificity of RAC retention in RAC-MISPE, RAC-NIP was also prepared and packed in a cartridge, and the same experiment previously performed on RAC-MISPE was made. In this case, 48.4% of RAC and 88.2% of DM-RAC were lost in the waste (loading and washing), suggesting a good imprinting effect of RAC-MISPE. For this reason, a further attempt was made using the RAC-MISPE cartridge and mimicking the real composition in terms of RAC and DM-RAC concentration obtained during the radiolabeling step of [^11^C]RAC production, as reported by Perkins and co-workers [6]. Thus, after the conditioning step, 1 mL of a solution containing 1000 µg mL^−1^ of DM-RAC and 10 µg mL^−1^ of RAC was loaded on the RAC-MISPE cartridge and treated using the same conditioning, washing, reconditioning, and elution processes mentioned above for RAC-MISPE cartridge.

**Figure 9:**
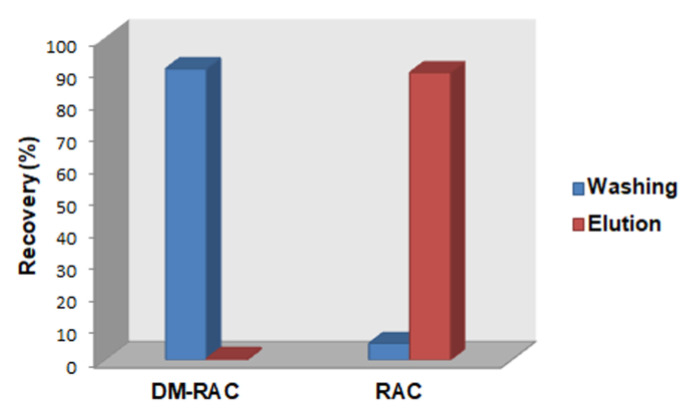
Recovery yield in the washing and elution steps obtained loading RAC-MISPE cartridge with a solution containing an excess of DM-RAC (1000 µg mL^−1^) compared to RAC (10 µg mL^−1^).

As can be seen in Figure 9, also in the presence of a large excess of precursor, a good recovery of unlabeled RAC (90%, RSD 4.6%) was possible. Indeed, during washing steps, 90.5% (RSD 5.2%) of DM-RAC was released, and only a little amount of RAC (0.5%, RSD 0.2%) was lost. However, in the elution step, conducted using 5 mL of a mixture of H_2_O and EtOH (*v*/*v*, 9/1), a small amount of precursor was still detected (5.1%, RSD 0.5%). The satisfactory results obtained prompted us to use RAC-MIP as a stationary phase to purify [^11^C]RAC in an in-flow trap and release process.

### 3.2. Testing of RAC-MISPE with [^11^C]RAC Solutions

In our radiochemical experiments, we first tested the capacity of the RAC-MISPE cartridge to trap and release [^11^C]RAC previously purified by standard single-pass HPLC (set-up A). This solution is already mostly deprived of DM-RAC precursor and, therefore, should represent a simpler matrix to be submitted to the polymer; however, it is worth noting that this product was in a matrix of 0.9% NaCl and EtOH (up to 5%), which is different from the DMSO-based matrix for which the RAC-MISPE was optimized. Moreover, in these experiments, we did not perform any NaOAc/EtOH washing given that the elution of DM-RAC was not needed; therefore, we only reconditioned the polymer with NaOH to allow elution of the desired product. We loaded both diluted (with H_2_O) and undiluted [^11^C]RAC, and we assayed for radioactivity the solutions at each step of loading, washing with H_2_O, reconditioning with NaOH, and elution with EtOH. In both cases, <0.5% was detected in the waste (i.e., loading, washing, and reconditioning), while, respectively, 49% and 65% of starting products remained stuck in the RAC-MIP, with the remaining activity found in the eluted product. However, the most relevant information was that the flow rate achievable through the polymer was very low using a nitrogen gas push of 5 bar, estimated as an average of <0.3 mL min^−1^; in fact, the whole process lasted ~1.5 h, which equates to ~4 halflives of ^11^C, leaving only ~5% of starting radioactivity still available. For this reason, when moving to purify the real radiolabeling mixture, we packed RAC-MIP (200 mg) into a Valco metal cartridge, which allows using HPLC-rated fittings and, therefore, is able to sustain higher pressures and flow rates. This arrangement (set-up B) allowed the polymer to be placed instead of the HPLC column and to use the same syringe-driven injection valve/loop and HPLC pump used for the standard procedure. To introduce in the 10 mL injection loop the reaction mixture, as well as the different solutions used for washing (H_2_O), cleaning (NaOAc/EtOH), reconditioning (NaOH), and elution (EtOH), we employed a secondary vial connected to the injector valve syringe drive and used the HPLC pump as volume metering tool. The whole process was remotely controlled by the Synthra software interface, and the operator retrieved the solutions exiting the cartridge after each step.

**Figure 10:**
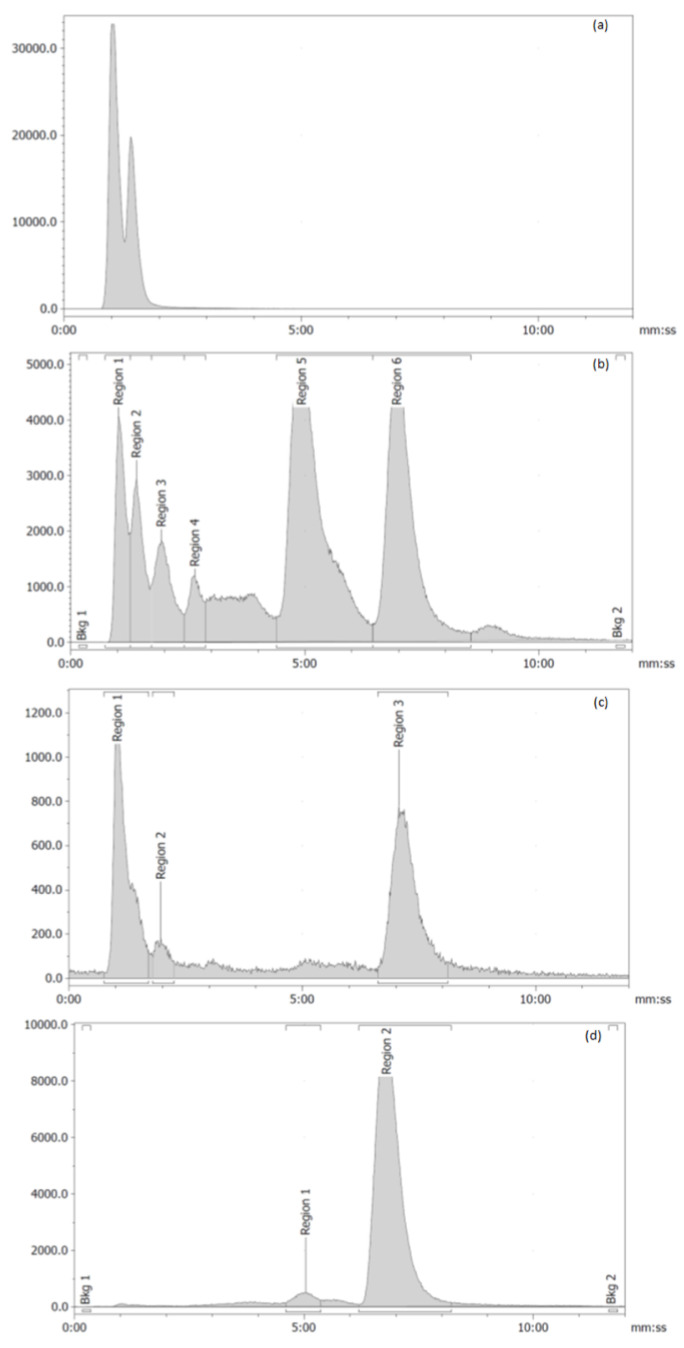
Radio-HPLC profiles of the fractions eluted out of (**a**) the MISPE. Loading and washing, (**b**) cleaning and (**c**) washing, reconditioning, (**d**) eluted product; (**a**–**c**) are considered “waste”. The radioactive peaks before 3 min are unreacted [^11^C]CH_3_I and [^11^C]CH_3_OH, the radioactive peak around 5 min is an unknown radioactive byproduct, and the radioactive peak around 7 min is the desired [^11^C]RAC.

In the first experiment using this set-up, the reaction mixture loading was performed at 0.5 mL min^−1^, and the HPLC pump experienced a backpressure of 160 bar, although the safety stop was also triggered due to a pressure spike (>500 bar) during this operation; in order not to have line or equipment breakages, the subsequent steps were performed at 0.3 mL min^−1^, thus slowing down the whole process. However, measuring the collected solutions, and comparing the radioactive quantities with the measurement from the GPextent, demonstrated that 30% of the radioactivity was collected in the eluted product fraction, while 67% was in the waste solutions, and the remainder 3% was estimated to be left in the RAC-MIP. The fractions collected were analyzed by HPLC, recording both radiochemical and UV adsorption profiles (220 nm). The radiochemical purity of the [^11^C]RAC in the eluted product fraction was 96%, therefore of acceptable quality for imaging use. The waste fractions also contained [^11^C]RAC in <50% amount, but these losses contributed to the overall lower recovery of the product in the final elution (Figure 10). Given the same analytical conditions employed, it was possible to compare the peak areas of the various fractions also using the UV channel. This allowed us to notice that the RAC peak was low in the first fractions (peak areas 77, 315, 298) and higher in the product fraction (1166), demonstrating the expected trend in selective trapping and release for RAC, although not as efficient as in the non-radioactive testing. On the other hand, DM-RAC had a peak area of 309 in the final product and was nearly completely eluted in the first fraction (peak area 2083), with smaller amounts eluted in the other fractions (peak area 440, 208). Moreover, this data demonstrated the expected trend in lack of retention of DM-RAC by the polymer, but in a less efficient manner than in the previous non-radioactive tests.

Unfortunately, the whole process lasted nearly 2 h, bringing down the activity yield to ~1% (i.e., vs. 30% radiochemical yield); for the same yield parameter, the standard process achieves a maximum of 6% in 25 min. Therefore, if compared, the process employing RAC imprinted polymer could provide better productivity if it were possible to reduce the processing times by employing faster flow rates.

With this target in mind, we modified the experiment by packing the RAC-MIP less tightly into the metal cartridge; this reduced the amount of polymer by approximately 1/3 compared to the previous experiment. However, this measure did not solve the backpressure issue, which remained at 150 bar or higher even at 0.4 mL min^−1^ flow rates; in addition, the reduced amount of RAC imprinted polymer in the cartridge did not allow successful trapping of [^11^C]RAC, which was in fact mostly eluted in the first fractions, with only 6% recovered in the product vial (vs. 30% of the previous experiment).

Still attempting to increase the flow rate and decrease the backpressure, we devised a different system (set-up C), in which we loaded the MIP into a glass Omnifit column with an adjustable packing bed. Compared to the Valco cartridge, this column has a wider diameter (25 mm and 10 mm vs. 4 mm) and should therefore allow higher flow rates with lower backpressure. We tested two different diameters of such columns, and we loaded them with the same amount of RAC-MIP (200 mg). As expected, we were able to realize higher flow rates without pressure spikes; in particular, we used 4 mL min^−1^ and 0.5 mL min^−1^ flow rates on the 25 mm-wide and 10 mm-wide columns, experiencing 5 bar and 3 bar, respectively. However, the inevitable looser packing, as well as the reduced depth of interaction with the support did not allow for an improved purification process. In the 30 min procedure employing the wider Omnifit column (25 mm diameter), most of the radioactivity (>60%) was recovered in the waste, while <5% was eluted as a product, demonstrating the fact that a slower flow rate or increased depth of interaction is desirable to allow efficient trapping of [^11^C]RAC. Aiming at not reducing the flow rate excessively, we used a narrower Omnifit column (10 mm diameter) that, at parity of loaded RAC-MIP, would increase the height of the support and, therefore, the number of longitudinal interactions. By doing this, in the 45 min purification process, we indeed recovered the capacity of the column to trap [^11^C]RAC, but we were not able to release it efficiently, as >80% of the radioactivity was estimated to be stuck in the RAC-MIP, and ~1% was recovered in the product fraction. This last phenomenon is aligned with our initial tests (set-up A), which basically feature a similar arrangement, for which we were not able to quantitatively recover the [^11^C]RAC from a previously purified solution.

## 4. Conclusions

In this work, we have synthesized and functionally characterized a new polymeric MISPE able to trap and release RAC, a molecule that, when labeled with ^11^C, represents one of the best PET tracers for D2 dopamine imaging. We optimized the operating conditions using non-radioactive standards and then tested its functionality in real radiochemical scenarios. In these latter experiments, we have realized that the MISPE provides high backpressures, which are indeed required to achieve the best trapping performance, but also efficient elution. We also realized that a reduced amount of RAC-MIP, as well as reduced longitudinal interactions, induces a reduction in trapping efficiency. Since such phenomena were not evident in the non-radioactive testing, we hypothesize that the peculiarly low trace levels of the radiochemical process require additional optimization strategies that cannot completely be anticipated without direct testing. One of the tested set-ups provided encouraging results, affording 96% pure product with a radiochemical yield of 30%; unfortunately, given the slow flow rate used to limit over-pressurization of the system, the process lasted 2 h, thus effectively providing an activity yield of ~1%. Future studies are required to improve on this result, potentially involving the use of longer metal cartridges and high-pressure fluidic equipment, so to ensure fast flow rates at high backpressures on tightly packed beds of RAC-MISPE.

## Data Availability

Not applicable.
